# Shoulder terminal sensory articular nerve radiofrequency ablation for nonsurgical refractory shoulder pain due to rotator cuff pathology and osteoarthritis: a technical note

**DOI:** 10.1093/pm/pnae035

**Published:** 2024-04-30

**Authors:** Taylor R Burnham, Scott Miller, Amanda N Cooper, Aaron Conger, Ameet S Nagpal, Max Eckmann, Zachary L McCormick

**Affiliations:** Department of Physical Medicine and Rehabilitation, University of Utah School of Medicine, Salt Lake City, UT, United States; Tennessee Orthopaedic Alliance, Nashville, TN, United States; Department of Physical Medicine and Rehabilitation, University of Utah School of Medicine, Salt Lake City, UT, United States; Department of Physical Medicine and Rehabilitation, University of Utah School of Medicine, Salt Lake City, UT, United States; Department of Orthopaedics & Physical Medicine, Medical University of South Carolina, Charleston, SC, United States; Department of Anesthesiology, University of Texas Health Science Center at San Antonio, TX, United States; Department of Physical Medicine and Rehabilitation, University of Utah School of Medicine, Salt Lake City, UT, United States

**Keywords:** Shoulder, Radiofrequency Ablation, Rotator Cuff, Osteoarthritis

## Abstract

**Background:**

Given the high prevalence of chronic shoulder pain and encouraging early results of terminal sensory articular branch radiofrequency ablation to treat shoulder pain, research is warranted to refine the procedural technique on the basis of updated neuroanatomic knowledge with the goal of further improving patient outcomes.

**Objective:**

We describe an updated radiofrequency ablation protocol that accounts for varied locations of the terminal sensory articular branches of the suprascapular, axillary, subscapular, and lateral pectoral nerves within individual patients.

**Design:**

Technical note.

**Methods:**

Cadaveric studies delineating the sensory innervation of the shoulder joint were reviewed, and a more comprehensive radiofrequency ablation protocol is proposed relative to historical descriptions.

**Conclusions:**

The proposed radiofrequency ablation protocol, which is based on neuroanatomic dissections of the shoulder joint, will provide a safe means of more complete sensory denervation and potentially improve clinical outcomes compared with historical descriptions, the efficacy of the new protocol must be confirmed in prospective studies.

## Introduction

Many individuals with degenerative shoulder pathology (ie, rotator cuff tendinosis/tearing, glenohumeral or acromial-clavicular arthritis, etc) experience chronic pain refractory to conservative treatments and unfortunately are also poor surgical candidates because of advanced age or medical comorbidities. Minimally invasive treatment options for this patient population are currently limited.

Advancements in radiofrequency ablation (RFA) techniques targeting peripheral structures offer a promising solution for nonsurgical management of intractable joint pain. For example, neuroanatomically based refinements to genicular RFA protocols improved sensory denervation in the knee joint, helping to establish this procedure as a safe and effective treatment option for patients with painful knee osteoarthritis.^1–5^ Using a similar approach, Eckmann et al. proposed a shoulder RFA (SRFA) technique targeting the terminal sensory articular branches (TSABs) of the suprascapular nerve (SN), axillary nerve (AN), and lateral pectoral nerve (LPN).^6^ Unfortunately, treatment with this technique was successful in fewer than half of patients.^7^ However, 5 of the 7 patients (71% [95% CI: 38%–105%]) who had rotator cuff pathology or osteoarthritis and were treated by the application of larger RFA lesions to the TSABs of the SN, AN, and LPN had >50% pain reduction after SRFA. As with genicular RFA, our understanding of glenohumeral joint innervation patterns is based on recent neuroanatomic dissections.^8^^,^^9^ As such, prospective research is warranted in better-defined patient populations with a more comprehensive SRFA protocol.

Here we outline a novel SRFA technique, describing how TSABs of the SN, AN, LPN, and upper subscapular nerve (USN) might be captured through the application of bipolar lesions to cover specific “target zones.” This description is intended to inform future investigations exploring the effectiveness of SRFA as a nonsurgical treatment for recalcitrant shoulder pain.

## Methods

### Patient preparation and SRFA procedure overview

The patient is prepared in typical sterile fashion and positioned accordingly (as will be discussed). The skin and superficial tissues are anesthetized with 1% preservative-free lidocaine before the SRFA introducer needle is inserted under fluoroscopic guidance at each target site. Care should be taken so the local anesthetic does not anesthetize the targeted nerves and hinder test stimulation. Before the ablation procedure, motor testing at 2 Hz up to 2.0 mV is performed at each site to minimize the likelihood of motor fiber capture. After a negative motor response, 1.0–1.5 mL of 2% lidocaine is injected via the side port of the introducer cannula to anesthetize the deep tissues for patient comfort.

All described procedures use 18-gauge multi-tined RFA probes with 5-mm active tips advanced in parallel and spaced no more than 10 mm apart to create bipolar strip lesions. This specific RFA probe allows coaxial probe advancement utilizing the recommended fluoroscopic views and produces a large pyramidal-shaped lesion distal to the RFA cannula. Additionally, bipolar strip lesioning is suggested to maximize lesion size and compensate for nerve variability. There are other RFA technologies reporting large lesions distal to the RFA cannula that could be used during the described technique. Conventional monopolar RFA probes should not be used with this technique because, when used perpendicularly, only a small lesion is made at the distal tip. In summary, providers should be familiar with RFA probe lesion morphologies and use them and other variables to increase RFA lesion size (needle gauge, lesion time, temperature) to maximize neural capture. We propose creating RFA lesions with a generator temperature setting of 80°C for 150 seconds each, but these parameters may be adjusted to change the lesion size.

### Fluoroscopically guided SRFA

Methods for capturing the following TSABs are based on radiographic techniques as described recently by Eckmann^8^ and Tran et al.^9^ We have provided “fluoroscopic suggestions” for C-arm positioning that could be helpful during setup. Though derived from previous studies, these suggestions are intended as supplemental guidance to assist with visualization. Our recommendation, and the basis of this technical piece, is to use the outlined target zones, which are defined by key, fixed anatomic landmarks. These landmarks should be the focus of needle placement for appropriate goal lesion zone capture with RFA technology, though it should be recognized that the probe angles and depths will vary with bony pathology and patient habitus and positioning.

#### Posterior zones (A and B)

**Figure 1.**
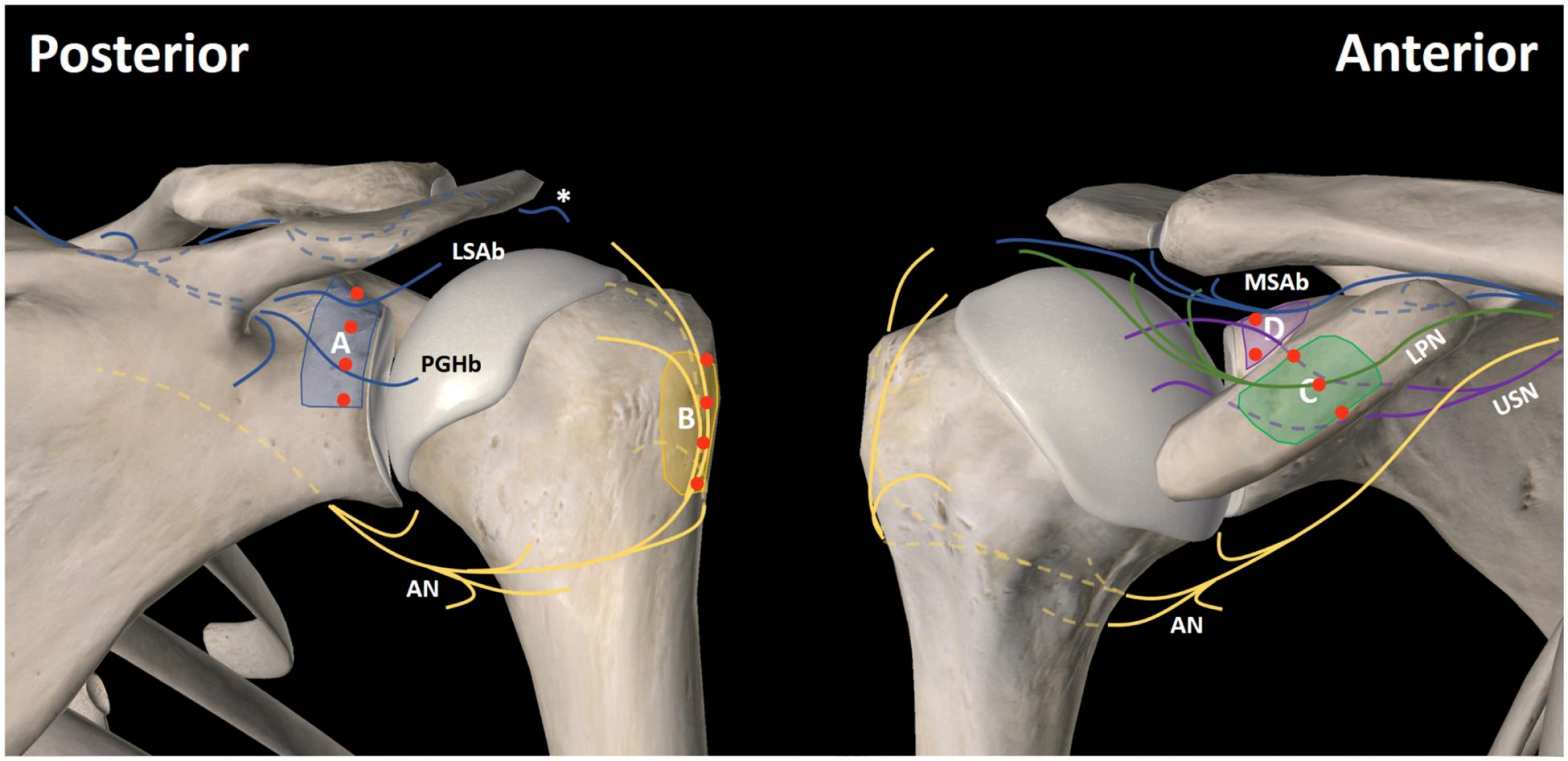
Four proposed target zones for fluoroscopically guided SRFA. Figure reproduced from Eckmann et al.^8^ Posterior approach: Zone A = suprascapular nerve (lateral subacromial branch [LSAb] and posterior glenohumeral branch [PGHb]); Zone B = axillary nerve (AN). Anterior Approach: Zone C = lateral pectoral nerve (LPN); Zone D = suprascapular nerve (medial subacromial branch [MSAb]) and upper subscapular nerve (USN). RFA electrode placements are represented by red circles. Bipolar RFA “strip lesions” will be created in order to traverse each respective target zone (A–D) in order to ensure capture of all intended terminal sensory articular branches shown.

***Patient positioning:*** To access the two posterior zones (Zone A and Zone B in Figure 1) and their constituent neural targets (2 of 3 sensory branches of the SN and the AN), the patient is placed in a prone position with the symptomatic shoulder internally rotated and the ipsilateral forearm pronated, such that the palm of the hand is facing the ceiling—or as close to this position as possible if the patient lacks full range of motion (eg, adhesive capsulitis).

**Figure 2.**
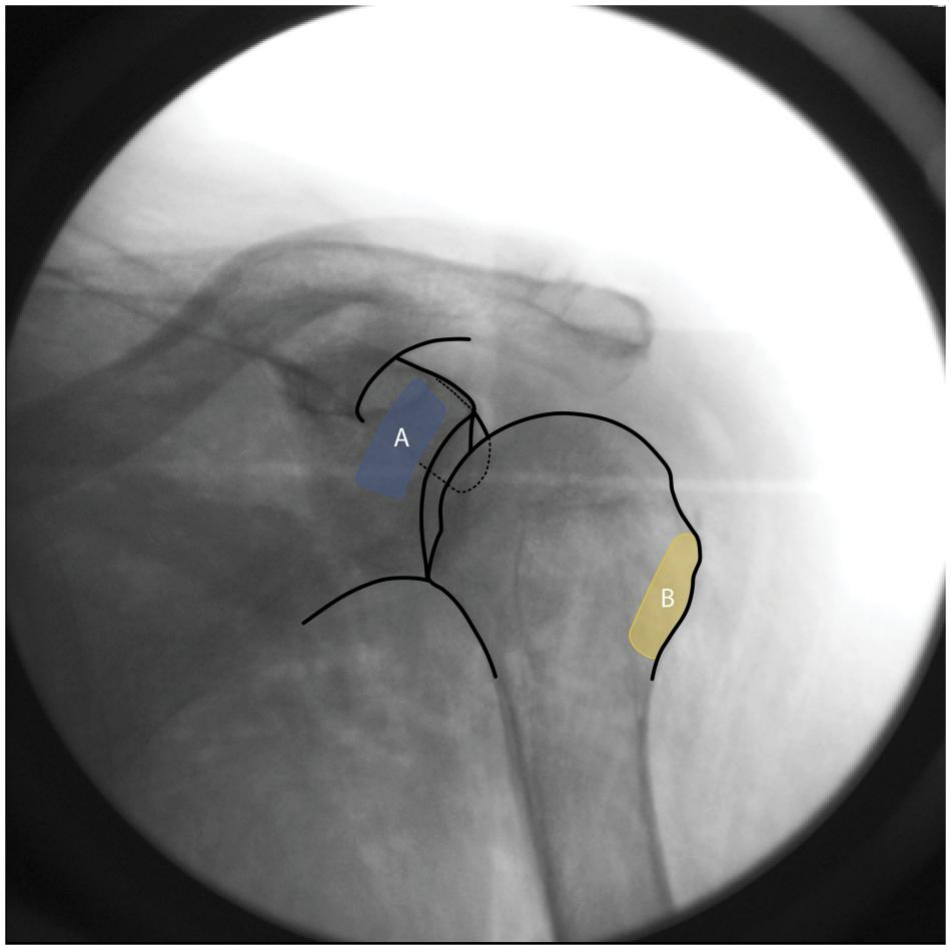
Fluoroscopic anatomy of key bony landmarks to target zones A and B.

***Key bony landmarks:*** Make fluoroscopic adjustments until the superior aspect of the coracoid process is aligned with the superior aspect of the glenoid rim and the lateral border of the coracoid neck is ∼1 cm medial to the glenohumeral joint line (Figure 2).

***Fluoroscopic suggestions:*** To obtain the appropriate view of the posterior border of the glenoid rim,^9^ ipsilaterally rotate the C-arm 15–45° away from midline with ∼15° of caudal tilt until a true view of the glenohumeral joint space is optimized.

##### Zone A: SN (lateral subacromial and posterior glenohumeral branches)

***Probe orientation:*** RFA probes are perpendicular to the posterior periosteal surface of the glenoid rim.

**Figure 3.**
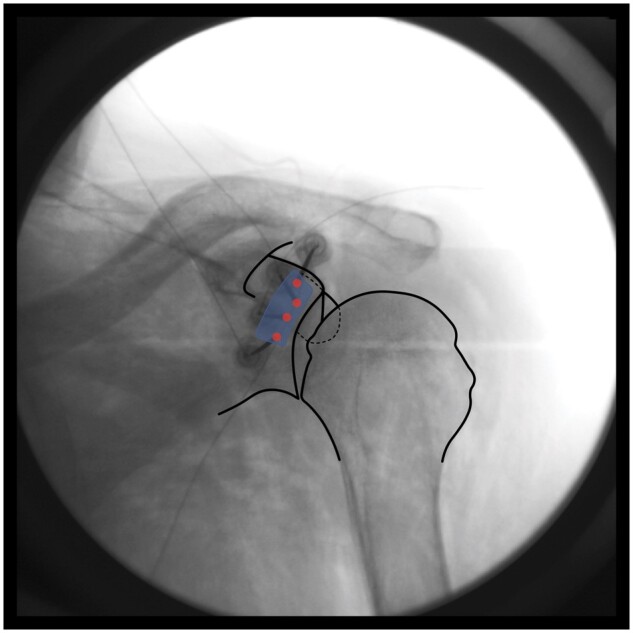
Bipolar strip lesion(s) to cover target zone A. RFA electrode placements are represented by red circles.

***Target zone and safety measures:*** The target zone is located in the upper half of the glenoid rim, medial to the glenoid rim to avoid the joint capsule/labrum, yet lateral to the spinoglenoid notch to avoid capturing motor fibers (Zone A in Figure 1). The superior lesions should be made at the lateral border of the coracoid neck. Once the probe is positioned, possible motor fiber involvement is assessed via motor testing, with monitoring for movement resulting from infraspinatus and supraspinatus muscle activation. After a negative motor stimulation response, lidocaine is injected, and a bipolar lesion is created. This process is repeated in order to form a “strip lesion” traversing the upper half of the glenoid rim. The number of bipolar lesions required for lateral subacromial branch and posterior glenohumeral branch ablation will vary with patient anatomy; however, we recommend applying as many lesions as necessary to cover the intended target zone, as outlined previously and as seen in Figure 3.

##### Zone B: AN (ascending humeral branches)

***Probe orientation:*** RFA probes are perpendicular to the posterolateral periosteal surface of the humeral head.

**Figure 4.**
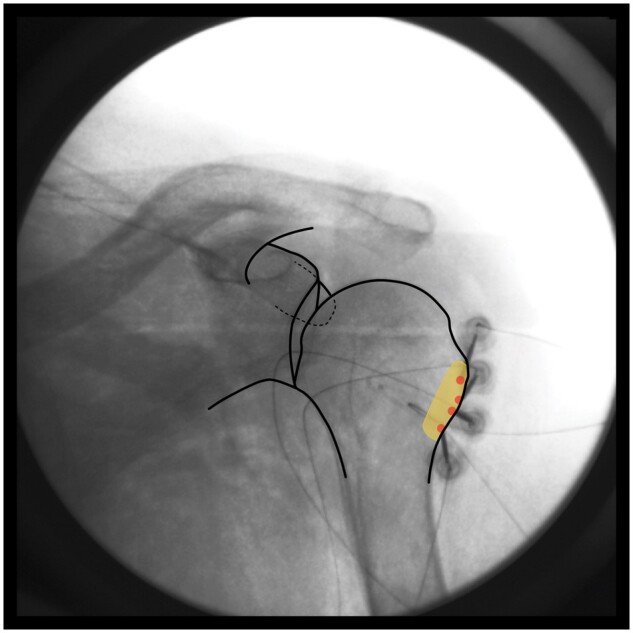
Bipolar strip lesion(s) to cover target zone B. RFA electrode placements are represented by red circles.

***Target zone and safety measures:*** The target zone consists of the lower two-thirds of the greater tubercle and humeral metaphysis, proximal to the surgical neck of the humerus (Zone B in Figure 1). After a negative motor stimulation response (no movement arising from deltoid, teres minor, or triceps muscle contractions), lidocaine is injected. Bipolar lesions are repeated as necessary to form a strip lesion traversing the interval between the lower two-thirds of the greater humeral tubercle and the surgical neck of the humerus, as shown in Figure 4.

#### Anterior zones (C and D)

***Patient positioning:*** To access the two anterior zones (Zone C and Zone D in Figure 1) and their constituent neural targets (LPN, 1 of 3 sensory branches of the SN, and the USN), the patient is placed in a supine position with the forearm on the symptomatic side resting supinated.

**Figure 5.**
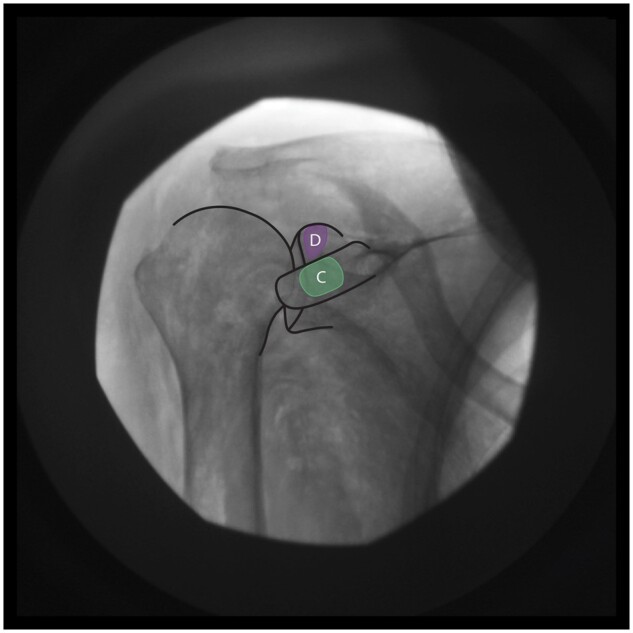
Fluoroscopic anatomy of key bony landmarks to target zones C and D.

***Key bony landmarks:*** Make fluoroscopic adjustments until the lateral one-third of the coracoid process extends just lateral to the glenohumeral joint line, and the superior aspect of the coracoid is positioned in the superior one-fourth of the glenohumeral joint line (Figure 5).

***Fluoroscopic suggestions:*** Introduce ∼15° of cephalad tilt and 15° of ipsilateral oblique rotation with C-arm adjustments to acquire the appropriate “thumbs-down” view of the coracoid process and to reveal access to the superior, anterior glenoid rim.

##### Zone C: LPN

***Probe orientation:*** RFA probes are perpendicular to the anterosuperior periosteal surface of the coracoid process.

**Figure 6.**
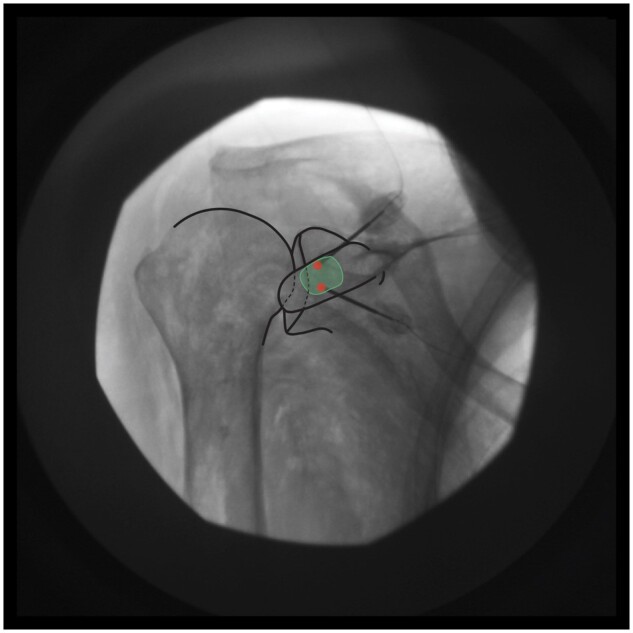
Bipolar strip lesion(s) to cover target zone C. RFA electrode placements are represented by red circles.

***Target zone and safety measures:*** The target zone is the midpoint of the length of the coracoid process (ie, 50% of the distance between the distal tip and the proximal border of the coracoid process), located just medial to the glenohumeral joint line. The RFA probes are placed along the short axis of the coracoid at this midpoint (Zone C in Figure 1). After a negative motor stimulation response, lidocaine is injected. At least two bipolar lesions are created to form a strip lesion traversing the anterosuperior portion of the coracoid process along its proximodistal midline, as shown in Figure 6.

##### Zone D: SN (medial subacromial branch) and USN

***Probe orientation:*** RFA probes are perpendicular to the anterior periosteal surface of the glenoid rim, just superior to the coracoid process and medial to the glenohumeral joint line.

**Figure 7.**
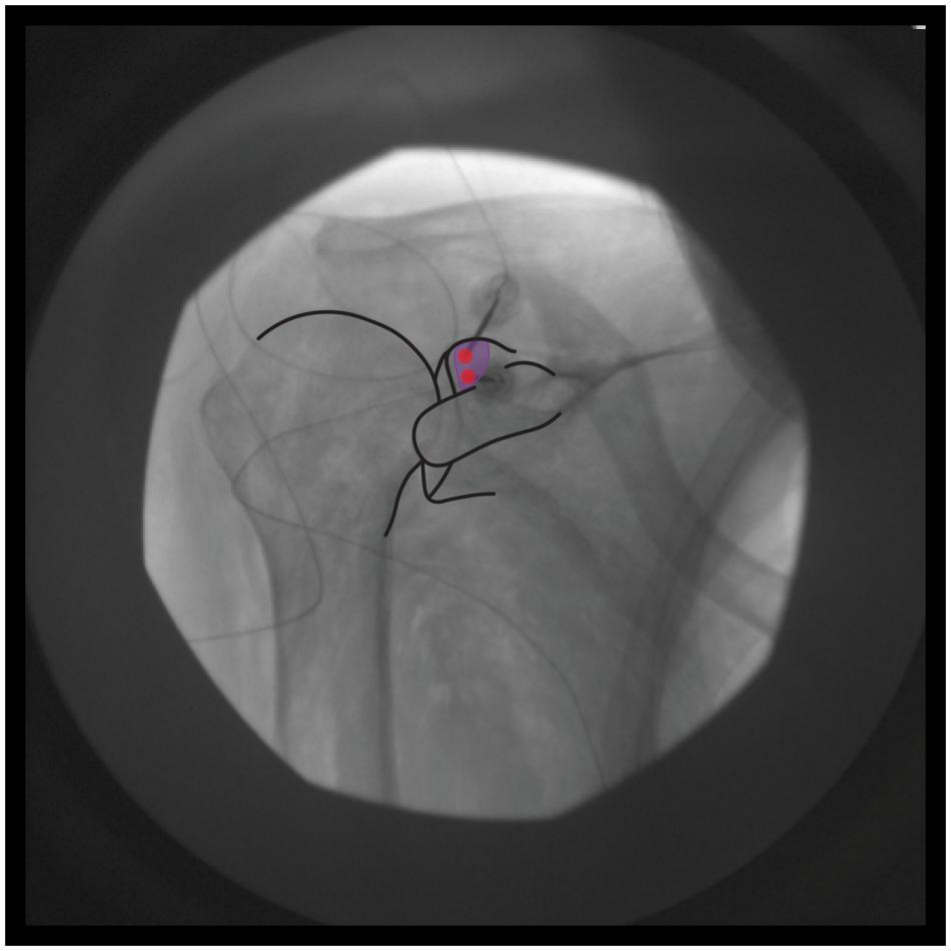
Bipolar strip lesion(s) to cover target zone D. RFA electrode placements are represented by red circles.

***Target zone and safety measures:*** The target zone is the superior one-fourth of the glenoid rim (Zone D in Figure 1). After confirmation of motor fiber exclusion, lidocaine is injected. At least two bipolar lesions are created to form a strip lesion that traverses Zone D, as shown in Figure 7.

## Discussion

Effectively capturing target nerves is arguably the most important determinant of RFA treatment success, with more comprehensive disruption of afferent nociceptive pathways leading to better outcomes. The bipolar strip lesions described here might more reliably capture TSABs, which have known inherent anatomic variability.^6^ This proposition builds on existing SRFA studies in which greater pain relief has been observed with larger SRFA lesions than with smaller, traditional lesions.^7^^,^^10^

Expanding the SRFA technique to capture additional nerves necessitates understanding the surrounding anatomy to prevent potential complications. Lying just deep to the LPN target zone on the coracoid process are important neurovascular structures, such as the brachial plexus, subclavian vessels, and the axillary artery, which could be violated if the RFA cannula were to unintentionally slip from its bony landmark. Bipolar strip lesions targeting the SN must be sufficiently medial to prevent joint capsule breach and possible labrum or biceps tendon damage, yet lateral to the spinoglenoid notch to avoid motor branch capture.
